# Secondhand Smoke and Biomass Fuel Exposure as Risk Factors for Pulmonary Tuberculosis: A Matched Case-Control Study From Southern Haryana

**DOI:** 10.7759/cureus.88118

**Published:** 2025-07-16

**Authors:** Abhishek Singh, Jayesh Singh, Neeraj Gour, Vipin Goyal

**Affiliations:** 1 Community Medicine, Shaheed Hasan Khan Mewati (SHKM) Government Medical College, Nuh, IND; 2 Medicine, University of Buckingham, Buckingham, GBR; 3 Respiratory Medicine, Shaheed Hasan Khan Mewati (SHKM) Government Medical College, Nuh, IND

**Keywords:** indoor air pollution, secondhand smoke, solid fuel smoke, tobacco, tuberculosis

## Abstract

Introduction

Secondhand smoke (SHS) exposure and biomass cooking fuel utilization represent persistent and growing health threats in regions where tuberculosis (TB) poses major public health risks. This comprehensive study conducted in Nuh district, Haryana, India, investigated the association between environmental exposures, including SHS and biomass cooking fuel use, with pulmonary TB development.

Methodology

This matched case-control study included 218 newly diagnosed pulmonary TB cases matched with 218 non-TB controls recruited from the same healthcare facility. Cases comprised nonsmoking adult men and women presenting as incident pulmonary TB patients diagnosed at the Tuberculosis Detection Center (TDC) through standard guidelines. Data were collected as a structured questionnaire. Bivariate logistic regression assessed associations between dependent and independent variables. Adjusted odds ratios were calculated for significant associations.

Results

Kitchen facilities analysis showed 192/436 (44%) homes without separate cooking areas and 215/436 (49.3%) lacking exhaust ventilation. Cooking fuel analysis revealed 73/218 (33.5%) cases used biomass fuels compared to 42/218 (19.3%) controls. Results demonstrated significant associations between TB and environmental risk factors: SHS exposure (adjusted odds ratio (OR) 2.83, 95% confidence interval (CI): 1.39-5.75), biomass fuel use (adjusted OR 1.85, 95% CI: 1.13-3.03), overcrowding (adjusted OR 2.85, 95% CI: 1.69-4.78), and inadequate ventilation (adjusted OR 1.65, 95% CI: 1.08-2.52).

Conclusions

The findings provide compelling evidence for the role of indoor air pollution and environmental tobacco smoke exposure in TB pathogenesis among vulnerable populations in resource-limited settings. SHS exposure and biomass cooking fuel use emerged as independent risk factors with substantial effect sizes, supporting biological mechanisms linking indoor air pollution to TB susceptibility. Additional environmental factors, including overcrowding, dampness, and inadequate ventilation, demonstrated strong associations with disease occurrence.

## Introduction

Tuberculosis (TB) remains a significant global health challenge, particularly in developing countries where environmental risk factors and socioeconomic determinants contribute substantially to disease burden [1]. The interplay between environmental exposures and susceptibility to TB has garnered increasing attention, with mounting evidence suggesting that inhaled pollutants, whether from active smoking, passive smoke exposure, or household air pollution from biomass combustion, significantly influence TB development and progression [2].

Secondhand smoke (SHS) exposure and biomass cooking fuel utilization represent persistent and growing health threats in regions where TB poses major public health risks [3]. In developing nations such as India, daily air pollution exposures from biomass cooking typically exceed established health guidelines by factors exceeding 20-fold, creating environments conducive to respiratory disease development. The physiological mechanisms underlying these associations involve the impairment of pulmonary defense systems, including alveolar macrophage phagocytic capacity, altered tumor necrosis factor-α and interleukin-1β production, compromised mucociliary clearance, and reduced T-cell proliferation, which are critical components of the innate immune response against Mycobacterium tuberculosis [4].

Cross-sectional epidemiological studies have evaluated various risk factors for the development of TB; however, limited research has specifically examined the causal relationship between passive smoking exposure and biomass fuel utilization in the Indian epidemiological context. The evidence linking cooking smoke exposure to TB remains tenuous despite established associations with other respiratory pathologies. Understanding these environmental determinants is essential for developing targeted public health interventions and informing evidence-based TB control strategies [5].

This study aimed to (1) assess the association between SHS exposure and pulmonary TB development, (2) evaluate the relationship between biomass cooking fuel use and TB risk, and (3) identify other environmental and sociodemographic risk factors for TB in this population. This investigation addresses critical knowledge gaps in understanding environmental TB risk factors in resource-limited settings where these exposures are prevalent.

## Materials and methods

Study design and setting

This matched case-control study was conducted at the Tuberculosis Detection Center (TDC) of Shaheed Hasan Khan Mewati (SHKM) Government Medical College in Nuh district, Haryana, India, between June 2023 and May 2024. The study region represents a predominantly rural, economically disadvantaged area with high TB burden and widespread use of traditional cooking fuels.

Study population and participant selection

The study included 218 newly diagnosed pulmonary TB cases and 218 age- and sex-matched non-TB controls. Cases comprised nonsmoking adult men and women presenting as incident pulmonary TB patients diagnosed at the TDC through standardized National Tuberculosis Elimination Program (NTEP) guidelines. All cases underwent sputum microscopy or culture confirmation according to established diagnostic protocols [6].

Individual matching (1:1 ratio) was performed for age (±5 years), gender, and rural area of residence to control for major confounding factors. Controls were selected using systematic random sampling from the TB screening register of individuals who tested negative for TB within the same period. Controls were nonsmoking individuals recruited from the same TB detection center among persons tested for but not diagnosed with TB. Exclusion criteria for both groups included individuals with asthma, diabetes mellitus, chronic obstructive pulmonary disease, lung cancer, HIV infection history, or those receiving long-term corticosteroid therapy.

Sample size calculation

Sample size determination utilized OpenEpi Software version 3.01 [7], calculating requirements at 80% statistical power and 95% two-sided confidence level. Considering 32.9% control exposure prevalence, 46.5% case exposure prevalence [3], and a 1:1 case-to-control ratio, the calculated sample size yielded 436 participants after continuity correction, comprising 218 cases and 218 matched controls. The calculated sample size provides 80% power for detecting the primary exposures. Secondary analyses were exploratory with adequate power (>70%) for detecting moderate effect sizes (odds ratio (OR) ≥2.0) for major environmental risk factors.

Data collection instrument

A comprehensive 23-element structured questionnaire incorporating both open-ended and closed-ended responses was developed, covering demographic and household characteristic domains. The instrument underwent rigorous translation and back-translation processes between English and Hindi to ensure semantic equivalence. Pilot testing among 20 subjects indicated an average completion time of 30-35 minutes, with a Cronbach's alpha value of the pilot sample of 0.85. Content validity was established through evaluation by 15 randomly selected faculty members for clarity, relevance, and acceptability (Appendix).

The questionnaire comprised two primary sections: demographic characteristics, including age, gender, education, occupation, religion, family type, residence, socioeconomic status, and household TB history; and household characteristics encompassing housing type, ownership, ventilation, overcrowding, dampness, cooking fuel types and usage duration, kitchen facilities, and smoking exposure patterns.

Some important operational definitions tabulated below were considered for this study (Table 1).

**Table 1:** Operational definitions.

Secondhand smoke (SHS) exposure	Defined for a non-smoker as exposure to tobacco smoke more than three times per week at home, work, or in public places
Overcrowding	Defined as more than two people per room in the household
Inadequate Ventilation	Defined as the absence of windows or an exhaust fan in the kitchen area
Dampness	Defined as the visible presence of damp patches or mold growth on the walls or ceiling
Smoking categorization	
Non-smoker	Individuals who never smoke or are exposed to tobacco smoke less than three times weekly
Active smoker	Current tobacco users or those discontinuing use within six months
Passive smoker	Non-smokers exposed to tobacco smoke more than three times weekly at home, work, or public places

Biomass fuel exposure included wood, dung, crop residues, and other organic materials used for cooking, while clean fuel comprised liquefied petroleum gas (LPG), electricity, and natural gas.

Statistical analysis

Data management utilized Microsoft Excel, with subsequent analysis performed using IBM SPSS Statistics Version 22.01 (IBM Corp., Armonk, NY). Categorical variables were presented as frequencies and percentages. Conditional logistic regression was used to account for the matched design. Bivariate analysis assessed associations between dependent and independent variables, with statistical significance defined as *P *< 0.05. Adjusted ORs with 95% confidence intervals (CIs) were calculated for significant associations.

Potential residual confounding from unmeasured factors such as nutritional status, genetic susceptibility, and precise exposure timing was acknowledged. Sensitivity analyses were performed, excluding participants with missing data.

Ethical considerations

Ethical approval (SHKM/IEC/22/37 dated October 14, 2022) was obtained from the Institutional Ethics Committee of the medical college. Written informed consent was obtained from all participants in their native language after explaining study procedures, risks, benefits, and voluntary participation. Participants were informed of their right to withdraw at any time without affecting their medical care. Data confidentiality was maintained through coded identifiers and secure data storage.

## Results

Demographic and socioeconomic characteristics

The study included 436 participants, comprising 218 TB cases and 218 matched controls. Male participants represented 236/436 (54.1%) of the study population, with 280/436 (64.2%) residing in rural areas. Age distribution showed median ages of 42.5 (32.0-55.0) years for cases versus 41.0 (31.0-53.0) years for controls, with 137/436 (31.4%) in the 30-44 years group and 145/436 (33.3%) in the 45-59 years group. Educational analysis revealed 192/436 (44.0%) illiteracy rates, while 221/436 (50.7%) belonged to lower socioeconomic strata. Religious composition included 360/436 (82.6%) Muslim participants, with 254/436 (58.3%) living in joint family structures. Household TB history within the preceding decade was reported by 57/436 (13.1%) of participants. Median household size was 6.0 (4.0-8.0) persons for cases versus 5.0 (4.0-7.0) persons for controls.

Comparative analysis revealed statistically significant differences between cases and controls for gender distribution (*P *< 0.001), age groups (*P *= 0.032), educational attainment (*P *< 0.001), and socioeconomic status (*P *= 0.003) (Table 2).

**Table 2:** Demographic and socioeconomic characteristics. *Statistical significance at *P *< 0.05.

Variable	Cases (*N *= 218), *n* (%)	Controls (*N *= 218), *n* (%)	Total (*N *= 436), *n* (%)	*χ*²-value	*P*-value
Gender					
Male	135 (61.9)	101 (46.3)	236 (54.1)	15.84	<0.001*
Female	83 (38.1)	117 (53.7)	200 (45.9)		
Age group (years)					
18-29	45 (20.6)	62 (28.4)	107 (24.5)	8.73	0.032*
30-44	78 (35.8)	59 (27.1)	137 (31.4)		
45-59	72 (33.0)	73 (33.5)	145 (33.3)		
≥60	23 (10.6)	24 (11.0)	47 (10.08)		
Education level					
Illiterate	115 (52.8)	77 (35.3)	192 (44.0)	22.45	<0.001*
Primary	58 (26.6)	69 (31.7)	127 (29.1)		
Secondary	35 (16.1)	52 (23.9)	87 (19.9)		
Higher secondary and above	10 (4.6)	20 (9.2)	30 (6.9)		
Socioeconomic status					
Lower	125 (57.3)	96 (44.0)	221 (50.7)	11.89	0.003*
Middle	73 (33.5)	89 (40.8)	162 (37.2)		
Upper	20 (9.2)	33 (15.1)	53 (12.2)		

Household and environmental characteristics

Housing assessment demonstrated that 89/436 (20.4%) of participants resided in kutcha structures. Of the participants, 350 out of 436 (80.3%) owned their residences. Architectural evaluation revealed 162/436 (37.2%) of houses lacked windows in living areas, with visible dampness or mold growth observed in 186/436 (42.7%) of dwellings. Kitchen facilities analysis showed 192/436 (44.0%) of homes without separate cooking areas and 215/436 (49.3%) lacking exhaust ventilation systems.

Cooking fuel analysis revealed 73/218 (33.5%) of cases used biomass fuels compared to 42/218 (19.3%) of controls, with 99/436 (22.7%) utilizing biomass for more than five years. SHS exposure affected 38/436 (8.7%) of participants, with significantly higher prevalence among cases (Table 3).

**Table 3:** Household and environmental characteristics. *Statistical significance at *P *< 0.05.

Variable	Cases (*N* = 218), *n* (%)	Controls (*N* = 218), *n* (%)	Total (*N *= 436), *n* (%)	*χ*²-value	*P*-value
Housing type					
Pucca	89 (40.8)	115 (52.8)	204 (46.8)	8.34	0.015*
Semi-pucca	76 (34.9)	67 (30.7)	143 (32.8)		
Kutcha	53 (24.3)	36 (16.5)	89 (20.4)		
Overcrowding					
Present	142 (65.1)	98 (45.0)	240 (55.0)	18.42	<0.001*
Absent	76 (34.9)	120 (55.0)	196 (45.0)		
Dampness/Mold					
Present	112 (51.4)	74 (33.9)	186 (42.7)	11.29	<0.001*
Absent	106 (48.6)	144 (66.1)	250 (57.3)		
Kitchen ventilation					
Adequate	98 (45.0)	123 (56.4)	221 (50.7)	7.23	0.007*
Inadequate	120 (55.0)	95 (43.6)	215 (49.3)		
Primary cooking fuel					
Biomass	73 (33.5)	42 (19.3)	115 (26.4)	12.15	0.001*
Clean fuel	145 (66.5)	176 (80.7)	321 (73.6)		
Secondhand smoke					
Exposed	28 (12.8)	10 (4.6)	38 (8.7)	8.91	0.003*
Not exposed	190 (87.2)	208 (95.4)	398 (91.3)		

Risk factor analysis

Multivariate conditional logistic regression analysis identified several significant associations with TB development. Male gender demonstrated an adjusted OR of 2.05 (95% CI: 1.27-3.30), while higher education showed protective effects with an adjusted OR of 0.21 (95% CI: 0.10-0.44). Environmental factors demonstrated strong associations, with overcrowding showing an adjusted OR of 2.85 (95% CI: 1.69-4.78; χ² = 18.42), dampness or mold growth yielding 2.22 (95% CI: 1.39-3.54; χ² = 11.29), and SHS exposure producing 2.83 (95% CI: 1.39-5.75; χ² = 8.91). Biomass fuel use demonstrated an adjusted OR of 1.85 (95% CI: 1.13-3.03; χ² = 5.67) (Table 4).

**Table 4:** Multivariate analysis of risk factors. Data presented as adjusted odds ratio (95% CI). Statistical significance determined using logistic regression. *P-value < 0.05 considered statistically significant. CI, confidence interval; OR, odds ratio

Variable	Adjusted OR	95% CI	Wald χ²	*P*-value
Male gender	2.05	1.27-3.30	8.45	0.004*
Higher education	0.21	0.10-0.44	15.23	<0.001*
Lower socioeconomic status	1.78	1.15-2.76	6.89	0.009*
Overcrowding	2.85	1.69-4.78	18.42	<0.001*
Dampness/Mold	2.22	1.39-3.54	11.29	<0.001*
Inadequate ventilation	1.65	1.08-2.52	5.34	0.021*
Biomass fuel use	1.85	1.13-3.03	5.67	0.017*
Secondhand smoke	2.83	1.39-5.75	8.91	0.003*

Dose-response analysis revealed increasing risk with exposure duration: biomass fuel use <5 years (OR 1.45, 95% CI: 0.87-2.41), 5-10 years (OR 1.72, 95% CI: 1.05-2.82), >10 years (OR 2.31, 95% CI: 1.38-3.87), *P*-value = 0.003.

## Discussion

This matched case-control study provides compelling evidence for the association between environmental exposures, particularly SHS and biomass cooking fuel use, with pulmonary TB development in a resource-limited setting. The findings contribute significantly to the growing body of literature documenting the role of indoor air pollution in TB pathogenesis and have important implications for public health policy and TB control strategies.

Environmental risk factors and TB susceptibility

The study's findings regarding biomass fuel use align with emerging evidence from multiple international investigations. Our results demonstrate that households using biomass fuels have 1.85-fold higher odds of pulmonary TB compared to those using clean fuels, consistent with previous research [8,9]. Our findings demonstrate a dose-response relationship with biomass fuel exposure duration: <5 years (OR 1.45, 95% CI: 0.87-2.41), 5-10 years (OR 1.72, 95% CI: 1.05-2.82), >10 years (OR 2.31, 95% CI: 1.38-3.87), *P*-value = 0.003. The biological plausibility of this association rests on the impairment of alveolar macrophage function caused by particulate matter and toxic compounds released during biomass combustion. These pollutants can compromise the innate immune response mechanisms critical for defending against Mycobacterium TB infection, including reduced phagocytic capacity and altered cytokine production patterns.

The association between SHS exposure and TB development represents a particularly important finding, with exposed individuals demonstrating nearly threefold increased odds of disease occurrence. Our SHS findings (OR 2.83) are higher than the pooled estimate from Lin et al.'s meta-analysis (OR 1.56, 95% CI: 1.24-1.96) but consistent with recent studies from high-burden settings and pediatric populations [10,11]. Jafta's systematic review and meta-analysis noted that TB disease in environmental tobacco smoke (ETS) studies produced a pooled OR of 2.8 (95% CI: 0.9-4.8), which was higher than the OR for TB infection (OR 1.9, 95% CI: 0.9-2.9) for children exposed to ETS compared to non-exposed children [11]. For biomass fuel use, our OR of 1.85 aligns with recent Indian data (OR 1.71, 95% CI: 1.07-2.38) from National Family Health Survey-5 (NFHS-5) analysis [12]. This relationship has been documented in studies by other authors supporting the biological mechanisms whereby tobacco smoke constituents impair respiratory defense systems [13,14]. The immunosuppressive effects of nicotine and other tobacco-derived compounds can reduce T-cell proliferation, impair macrophage activation, and compromise the cellular immune response essential for TB control.

Sociodemographic determinants and disease risk

The study's demographic findings reveal important patterns consistent with global TB epidemiology. Male predominance among cases reflects documented gender disparities in TB occurrence, potentially attributable to occupational exposures, social behaviors including tobacco and alcohol use, and differential healthcare-seeking patterns. The protective effect of higher education likely operates through multiple pathways, including improved health literacy, better access to healthcare services, enhanced nutritional status, and reduced exposure to environmental risk factors.

Socioeconomic status emerged as a critical determinant, with lower-income households demonstrating significantly higher TB risk. This association reflects the complex interplay between poverty and TB transmission, encompassing factors such as overcrowding, malnutrition, delayed healthcare access, and increased exposure to environmental pollutants. These findings are consistent with research by previous researchers emphasizing the fundamental role of social determinants in TB epidemiology [3,15].

Housing and environmental conditions

The study's architectural and environmental findings highlight the importance of housing quality in TB prevention. Overcrowding demonstrated the strongest association with disease occurrence (χ² = 18.42, *P *< 0.001), likely facilitating airborne transmission through increased contact density and reduced air circulation. The presence of dampness and mold growth (χ² = 11.29, *P *< 0.001) indicates poor housing conditions that may compromise respiratory health and increase susceptibility to infectious diseases.

Kitchen characteristics, including the absence of separate cooking areas and inadequate ventilation, emerged as significant risk factors. These findings support previous research demonstrating elevated particulate matter concentrations in poorly ventilated cooking environments, as documented by others [16,17]. The concentration of cooking smoke in living areas increases exposure duration and intensity, potentially exacerbating respiratory health impacts.

Clinical and public health implications

These findings have substantial implications for TB control strategies in resource-limited settings. The identification of modifiable environmental risk factors suggests opportunities for targeted interventions that could reduce TB incidence at the population level [18]. Clean cooking fuel promotion programs, improved housing ventilation systems, and tobacco control initiatives represent potential intervention strategies with demonstrated feasibility in similar settings [19].

The strong associations observed suggest that environmental modification strategies could serve as effective adjuncts to traditional TB control measures. Integration of environmental health interventions with existing TB programs could enhance overall program effectiveness and contribute to sustainable disease burden reduction. These interventions align with global initiatives promoting clean cooking technologies and indoor air quality improvement as essential components of comprehensive health development strategies [20].

Strengths of the study

The study demonstrates several notable strengths, including matched case-control design with appropriate sample size calculation, comprehensive assessment of multiple environmental risk factors, standardized exposure definitions, and rigorous questionnaire development with translation validation. The inclusion of diverse socioeconomic and environmental variables provides a holistic perspective on TB risk factors in the study population. 

Study limitations

Several limitations warrant consideration: (1) Recall bias may affect exposure assessment accuracy, potentially leading to differential misclassification between cases and controls, (2) selection bias possible due to hospital-based recruitment may limit generalizability, (3) exposure misclassification likely due to self-reporting without objective measurements such as personal PM2.5 monitors or biomarkers, (4) residual confounding from unmeasured factors including nutritional status, genetic susceptibility, and precise exposure timing, (5) cross-sectional exposure assessment may not capture lifetime exposure patterns or temporal relationships, and (6) generalizability limited to similar rural populations in Northern India. The reliance on self-reported exposure assessment may introduce recall bias and exposure misclassification.

Recommendations

Future studies should incorporate objective exposure measurements, including personal monitoring devices for PM2.5 and carbon monoxide, and other biomarkers for tobacco smoke exposure.

## Conclusions

This matched case-control study provides robust evidence for significant associations between environmental exposures and pulmonary TB development in a resource-limited setting. SHS exposure and biomass cooking fuel use emerged as independent risk factors with substantial effect sizes, supporting biological mechanisms linking indoor air pollution to TB susceptibility. Additional environmental factors, including overcrowding, dampness, and inadequate ventilation, demonstrated strong associations with disease occurrence. Implementation of clean cooking fuel programs, improved housing ventilation systems, and tobacco control initiatives could contribute significantly to TB prevention efforts in similar epidemiological settings.

## Appendices

Appendix 

**Table 5:** Study questionnaire.

Structured Questionnaire for Data Collection	
23-element questionnaire used for demographic and household characteristic assessment	
Demographic profile	
Age (in completed years)	
Gender:	Male/Female
Highest educational qualification:	Illiterate/Primary/Secondary/Higher Secondary/Graduate/Post-graduate
Occupation:	Student/Housewife/Agriculture/Business/Service/Labor/Unemployed
		Religion:	Hindu/Muslim/Sikh/Christian/Others
Family type:	Nuclear/Joint/Extended
Area of residence:	Rural/Urban
Socioeconomic status:	Lower/Middle/Upper (Based on modified Kuppuswamy scale)
Family history of TB in last 10 years:	Yes/No
Housing Characteristics	
Type of house:	Pucca/Semi-pucca/Kutcha
Ownership of house:	Own/Rented/Others
Number of rooms in house:	Open-ended
Number of persons living in house:	Open-ended
Presence of windows in living room:	Yes/No
Presence of dampness/mold in house:	Yes/No
Separate kitchen available:	Yes/No
Kitchen ventilation:	Adequate/Inadequate
Cooking Fuel and Smoking	
Primary cooking fuel used:	LPG/Electricity/Wood/Dung/Crop residue/Kerosene/Others
Duration of biomass fuel use (years):	<1/1-5/>5
Smoking status of household members:	Never smoker/Ex-smoker/Current smoker
Frequency of passive smoke exposure:	Never/<3 times per week/≥3 times per week
Location of passive smoke exposure:	Home/Workplace/Public places/Multiple locations
Duration of passive smoke exposure: (years)	<1/1-5/>5
